# *N* ^2^,*N* ^2′^-Bis(2-hydroxy­benzyl­idene)-2,2′-bipyridyl-3,3′-dicarbohydrazide

**DOI:** 10.1107/S1600536808038087

**Published:** 2008-12-10

**Authors:** Shao-Bin Miao, Lu-Lu Zang, Ya-Wei Fan, Bao-Ming Ji

**Affiliations:** a College of Chemistry and Chemical Engineering, Luoyang Normal University, Luoyang 471022, People’s Republic of China

## Abstract

In the title compound, C_26_H_20_N_6_O_4_, the two aroylhydrazone side groups exist as diastereomeres, both in the keto form in the crystal structure. The aroylhydrazone units support the mol­ecular conformation through an intra­molecular N—H⋯O hydrogen bond. Two mol­ecules are connected into a centrosymmetric dimer by inter­molecular N—H⋯N hydrogen bonds. These dimers are connected into chains along the *a* axis by inter­molecular O—H⋯O hydrogen bonds. The combination of these hydrogen bonds results in layers in the *bc* plane. The layers are further linked by weak C—H⋯π contacts to form a three-dimensional network structure.

## Related literature

For syntheses, structures and ligand conformations of Ag^I^ complexes with flexible *N*,*N*′-di(2-pyrid­yl)adipoamide ligands, see: Chen *et al.* (2007 ▶). For palladium-catalysed allylic alkyl­ation using chiral hydrazones as ligands, see: Mino *et al.* (2001 ▶). For the biological activity of hydrazones and their metal complexes, see: Rodriguez-Argüelles *et al.* (2004 ▶); Wiley & Clevenger (1962 ▶). For coordinated hydrazone ligands as nucleophiles, see: Wood *et al.* (2004 ▶). For a new fluorescent rhodamine hydrazone chemosensor for Cu^II^, see: Xiang *et al.* (2006 ▶).
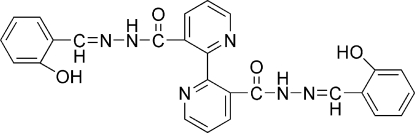


## Experimental

### 

#### Crystal data

C_26_H_20_N_6_O_4_
*M* *_r_* = 480.48
Triclinic, 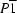

*a* = 9.4251 (13) Å
*b* = 11.7642 (16) Å
*c* = 12.0384 (16) Å
α = 98.842 (2)°
β = 108.895 (2)°
γ = 104.591 (2)°
*V* = 1181.1 (3) Å^3^
*Z* = 2
Mo *K*α radiation
μ = 0.10 mm^−1^
*T* = 293 (2) K
0.37 × 0.25 × 0.10 mm

#### Data collection

Bruker SMART CCD area-detector diffractometer
Absorption correction: multi-scan (*SADABS*; Sheldrick, 1996 ▶) *T*_min_ = 0.956, *T*_max_ = 0.991
8593 measured reflections
4281 independent reflections
3119 reflections with *I* > 2σ(*I*)
*R*_int_ = 0.021

#### Refinement

*R*[*F* ^2^ > 2σ(*F* ^2^)] = 0.041
*wR*(*F* ^2^) = 0.111
*S* = 1.02
4281 reflections
327 parameters
H-atom parameters constrained
Δρ_max_ = 0.46 e Å^−3^
Δρ_min_ = −0.19 e Å^−3^

### 

Data collection: *SMART* (Bruker, 1999 ▶); cell refinement: *SAINT* (Bruker, 1999 ▶); data reduction: *SAINT*; program(s) used to solve structure: *SHELXS97* (Sheldrick, 2008 ▶); program(s) used to refine structure: *SHELXL97* (Sheldrick, 2008 ▶); molecular graphics: *SHELXTL* (Sheldrick, 2008 ▶); software used to prepare material for publication: *SHELXTL* and *PLATON* (Spek, 2003 ▶).

## Supplementary Material

Crystal structure: contains datablocks global, I. DOI: 10.1107/S1600536808038087/si2129sup1.cif

Structure factors: contains datablocks I. DOI: 10.1107/S1600536808038087/si2129Isup2.hkl

Additional supplementary materials:  crystallographic information; 3D view; checkCIF report

## Figures and Tables

**Table 1:** Hydrogen-bond geometry (Å, °) *Cg*3 and *Cg*4 are the centroids of the benzene rings C1–C6 and C21–C26, respectively.

*D*—H⋯*A*	*D*—H	H⋯*A*	*D*⋯*A*	*D*—H⋯*A*
N5—H5*D*⋯O2	0.86	2.15	2.962 (2)	157
N2—H2*D*⋯N4^i^	0.86	2.17	2.985 (2)	159
O4—H4⋯O3^ii^	0.82	1.92	2.736 (2)	172
O1—H1⋯N1	0.82	1.95	2.663 (2)	145
C10—H10⋯*Cg*3^iii^	0.93	2.76	3.458 (2)	133
C11—H11⋯*Cg*4^iv^	0.93	2.73	3.588 (2)	154

Symmetry codes: (i) 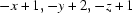; (ii) 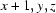; (iii) 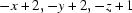; (iv) 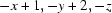.

**Fig. 1.**
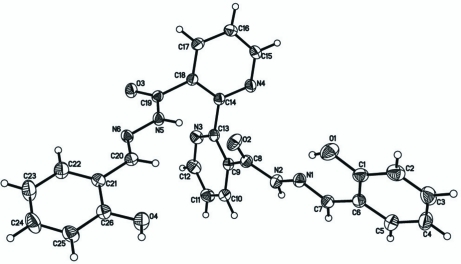
ORTEP drawing (30% probability displacement ellipsoids) of a single molecule of the title compound.

**Fig. 2.**
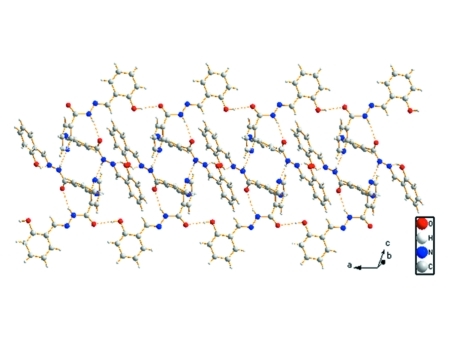
Unit cell packing diagram for the title compound. Hydrogen bonds are shown with dashed lines.

**Fig. 3.**
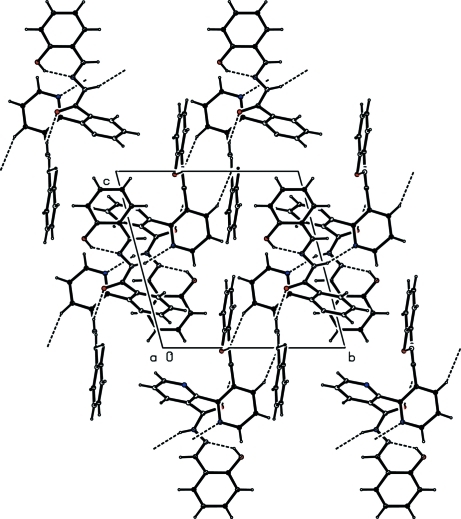
A section of the layered structure viewed down the a axis.

## supplementary crystallographic information

### Comment

Hydrazones and their metal complexes have gained a special attraction due to their biological activity (Rodriguez-Argüelles *et al.*, 2004; Wiley & Clevenger, 1962). These compounds have also been proposed as chemosensors (Xiang *et al.*, 2006), catalysts (Mino *et al.*, 2001) and nucleophiles (Wood *et al.*, 2004). Hydrazone ligands can coordinate with metal ions to produce stable metal complexes owing to their facile keto–enol tautomerism.

As shown in Fig. 1, two aroylhydrazone units are situated on both sides of the 2,2'-dipyridyl linking group which can decrease the steric hindrance among the pyridyl rings. The two aroylhydrazone side groups exist as diastereomeres, both in the keto form in the crystal structure. The aroylhydrazone units support the molecular conformation through an intramolecular N—H···O hydrogen bond (Table 1).

The dihedral angle between two pyridine rings of the 2,2'-dipyridyl group is 105.26 (2)°. The bond distances and angles are all in normal ranges. The distances of the C8—O2, C19—O3, N1—C7 and N6—C20 are 1.226 (2), 1.229 (2), 1.279 (2) and 1.280 (2) Å, respectively, which have the features of typical C═O and C═N double bonds (Chen *et al.*, 2007). This confirms that the compound exists in the keto form.

A pair of intermolecular N—H···N hydrogen bonds connect two adjacent molecules into dimers *via* inversion centres (Fig. 2). These dimers are connected into chains along *a* axis by intermolecular O4—H4···O3 hydrogen bonds. The combination of both hydrogen bonds generate layers which extend along the *b*+*c* direction (Fig. 3). The layers are linked by weak C—H···π contacts (Table 1) to form a three-dimensional network structure. Cg3 and Cg4 are the centroids of the benzene rings C1–C6 and C21–C26, respectively. There is another intramolecular hydrogen bond, O1—H1···N1, which results from the planar geometry in the H1–O1–C1–C6–C7–N1 ring system (Table 1).

### Experimental

A mixture of 2,2'-bipyridyl-3,3'-diformylhydrazide (0.272 g, 1 mmol), salicylaldehyde (2.5 mmol, 0.26 ml) and a drop of glacial acetic acid in ethanol (20 ml) was stirred at reflux temperature for 3 h. The solution was filtered and the filtrate was set aside to be crystallized. Yellow crystals suitable for the X-ray diffraction study were obtained after 5 d.

### Refinement

All of the non-hydrogen atoms were refined anisotropically. The hydrogen atoms were assigned with common isotropic displacement factors *U*_iso_(H) = 1.2*U*_eq_(C,N) and 1.5*U*_eq_(O), respectively, and included in the final refinement by using geometrical restraints, with C—H, N—H and O—H distances of 0.93, 0.86 and 0.82 Å.

### Crystal data

**Table:**

C_26_H_20_N_6_O_4_	*Z* = 2
*M**_r_* = 480.48	*F*(000) = 500
Triclinic, *P*1	*D*_x_ = 1.351 Mg m^−^^3^
*a* = 9.4251 (13) Å	Mo *K*α radiation, λ = 0.71073 Å
*b* = 11.7642 (16) Å	Cell parameters from 2245 reflections
*c* = 12.0384 (16) Å	θ = 2.4–23.8°
α = 98.842 (2)°	µ = 0.10 mm^−^^1^
β = 108.895 (2)°	*T* = 293 K
γ = 104.591 (2)°	Block, yellow
*V* = 1181.1 (3) Å^3^	0.37 × 0.25 × 0.10 mm

### Data collection

**Table:**

Bruker SMART CCD area-detector diffractometer	4281 independent reflections
Radiation source: fine-focus sealed tube	3119 reflections with *I* > 2σ(*I*)
graphite	*R*_int_ = 0.021
φ and ω scans	θ_max_ = 25.5°, θ_min_ = 2.4°
Absorption correction: multi-scan (*SADABS*; Sheldrick, 1996)	*h* = −11→11
*T*_min_ = 0.956, *T*_max_ = 0.991	*k* = −14→14
8593 measured reflections	*l* = −14→14

### Refinement

**Table:**

Refinement on *F*^2^	Primary atom site location: structure-invariant direct methods
Least-squares matrix: full	Secondary atom site location: difference Fourier map
*R*[*F*^2^ > 2σ(*F*^2^)] = 0.041	Hydrogen site location: inferred from neighbouring sites
*wR*(*F*^2^) = 0.111	H-atom parameters constrained
*S* = 1.02	*w* = 1/[σ^2^(*F*_o_^2^) + (0.0497*P*)^2^ + 0.2276*P*] where *P* = (*F*_o_^2^ + 2*F*_c_^2^)/3
4281 reflections	(Δ/σ)_max_ < 0.001
327 parameters	Δρ_max_ = 0.46 e Å^−^^3^
0 restraints	Δρ_min_ = −0.19 e Å^−^^3^

### Special details

**Table:**

Geometry. All e.s.d.'s (except the e.s.d. in the dihedral angle between two l.s. planes) are estimated using the full covariance matrix. The cell e.s.d.'s are taken into account individually in the estimation of e.s.d.'s in distances, angles and torsion angles; correlations between e.s.d.'s in cell parameters are only used when they are defined by crystal symmetry. An approximate (isotropic) treatment of cell e.s.d.'s is used for estimating e.s.d.'s involving l.s. planes.
Refinement. Refinement of *F*^2^ against ALL reflections. The weighted *R*-factor *wR* and goodness of fit *S* are based on *F*^2^, conventional *R*-factors *R* are based on *F*, with *F* set to zero for negative *F*^2^. The threshold expression of *F*^2^ > σ(*F*^2^) is used only for calculating *R*-factors(gt) *etc*. and is not relevant to the choice of reflections for refinement. *R*-factors based on *F*^2^ are statistically about twice as large as those based on *F*, and *R*-factors based on ALL data will be even larger.

### Fractional atomic coordinates and isotropic or equivalent isotropic displacement parameters (Å^2^)

**Table:**

	*x*	*y*	*z*	*U*_iso_*/*U*_eq_	
O1	0.91934 (18)	0.72851 (13)	0.61871 (15)	0.0628 (4)	
H1	0.8614	0.7551	0.5702	0.094*	
O2	0.54744 (16)	0.76483 (12)	0.33652 (13)	0.0531 (4)	
O3	0.03313 (14)	0.63641 (12)	0.00365 (11)	0.0452 (3)	
O4	0.76743 (15)	0.68107 (15)	0.01770 (12)	0.0586 (4)	
H4	0.8519	0.6749	0.0170	0.088*	
N1	0.81685 (17)	0.90089 (14)	0.53315 (14)	0.0400 (4)	
N2	0.70967 (17)	0.94879 (14)	0.46351 (13)	0.0399 (4)	
H2D	0.7280	1.0261	0.4795	0.048*	
N3	0.20060 (19)	0.93570 (15)	0.21026 (14)	0.0458 (4)	
N4	0.23711 (19)	0.79268 (14)	0.42134 (13)	0.0413 (4)	
N5	0.29163 (16)	0.65006 (13)	0.09343 (13)	0.0370 (4)	
H5D	0.3693	0.6630	0.1609	0.044*	
N6	0.31480 (17)	0.62971 (13)	−0.01446 (13)	0.0355 (4)	
C1	1.0361 (2)	0.82252 (18)	0.70788 (18)	0.0448 (5)	
C2	1.1409 (3)	0.7965 (2)	0.8036 (2)	0.0579 (6)	
H2	1.1310	0.7160	0.8046	0.070*	
C3	1.2592 (3)	0.8884 (2)	0.8969 (2)	0.0645 (7)	
H3	1.3274	0.8694	0.9612	0.077*	
C4	1.2783 (3)	1.0079 (2)	0.8970 (2)	0.0607 (6)	
H4A	1.3601	1.0695	0.9598	0.073*	
C5	1.1754 (2)	1.03568 (19)	0.80321 (18)	0.0488 (5)	
H5	1.1873	1.1167	0.8038	0.059*	
C6	1.0531 (2)	0.94455 (17)	0.70696 (16)	0.0383 (4)	
C7	0.9401 (2)	0.97971 (17)	0.61685 (16)	0.0385 (4)	
H7	0.9575	1.0617	0.6199	0.046*	
C8	0.5773 (2)	0.87486 (17)	0.37108 (16)	0.0375 (4)	
C9	0.4686 (2)	0.93978 (16)	0.31156 (15)	0.0348 (4)	
C10	0.5244 (2)	1.05037 (17)	0.28697 (17)	0.0423 (5)	
H10	0.6328	1.0889	0.3118	0.051*	
C11	0.4186 (3)	1.10226 (19)	0.22581 (19)	0.0509 (5)	
H11	0.4541	1.1773	0.2107	0.061*	
C12	0.2594 (3)	1.04164 (19)	0.18726 (19)	0.0518 (5)	
H12	0.1885	1.0759	0.1428	0.062*	
C13	0.3044 (2)	0.88770 (16)	0.27334 (15)	0.0340 (4)	
C14	0.22940 (19)	0.77607 (16)	0.30632 (15)	0.0332 (4)	
C15	0.1631 (2)	0.69644 (19)	0.45134 (18)	0.0488 (5)	
H15	0.1702	0.7059	0.5313	0.059*	
C16	0.0775 (2)	0.58480 (19)	0.37127 (18)	0.0514 (5)	
H16	0.0274	0.5208	0.3964	0.062*	
C17	0.0671 (2)	0.56921 (18)	0.25305 (18)	0.0459 (5)	
H17	0.0075	0.4950	0.1963	0.055*	
C18	0.14672 (19)	0.66555 (16)	0.21967 (15)	0.0334 (4)	
C19	0.1497 (2)	0.65002 (15)	0.09467 (16)	0.0333 (4)	
C20	0.4561 (2)	0.63447 (16)	−0.00311 (16)	0.0359 (4)	
H20	0.5342	0.6531	0.0740	0.043*	
C21	0.4959 (2)	0.61090 (16)	−0.11018 (16)	0.0358 (4)	
C22	0.3798 (2)	0.5622 (2)	−0.22627 (19)	0.0532 (5)	
H22	0.2738	0.5441	−0.2363	0.064*	
C23	0.4176 (3)	0.5402 (2)	−0.3266 (2)	0.0697 (7)	
H23	0.3381	0.5068	−0.4036	0.084*	
C24	0.5753 (3)	0.5682 (2)	−0.3121 (2)	0.0662 (7)	
H24	0.6017	0.5546	−0.3798	0.079*	
C25	0.6928 (2)	0.6157 (2)	−0.1985 (2)	0.0522 (5)	
H25	0.7985	0.6345	−0.1897	0.063*	
C26	0.6548 (2)	0.63589 (16)	−0.09660 (17)	0.0385 (4)	

### Atomic displacement parameters (Å^2^)

**Table:**

	*U*^11^	*U*^22^	*U*^33^	*U*^12^	*U*^13^	*U*^23^
O1	0.0565 (10)	0.0435 (9)	0.0758 (11)	0.0107 (7)	0.0136 (8)	0.0147 (8)
O2	0.0472 (8)	0.0374 (8)	0.0581 (9)	0.0142 (6)	0.0026 (7)	0.0033 (7)
O3	0.0298 (7)	0.0663 (9)	0.0356 (7)	0.0159 (6)	0.0098 (6)	0.0071 (6)
O4	0.0326 (7)	0.0918 (12)	0.0490 (9)	0.0242 (8)	0.0154 (7)	0.0044 (8)
N1	0.0359 (9)	0.0441 (9)	0.0378 (9)	0.0142 (7)	0.0103 (7)	0.0102 (7)
N2	0.0383 (9)	0.0361 (8)	0.0381 (9)	0.0111 (7)	0.0074 (7)	0.0068 (7)
N3	0.0452 (9)	0.0520 (10)	0.0467 (10)	0.0221 (8)	0.0181 (8)	0.0186 (8)
N4	0.0500 (10)	0.0399 (9)	0.0317 (8)	0.0123 (7)	0.0153 (7)	0.0064 (7)
N5	0.0292 (8)	0.0504 (9)	0.0291 (8)	0.0124 (7)	0.0101 (6)	0.0065 (7)
N6	0.0335 (8)	0.0437 (9)	0.0326 (8)	0.0135 (7)	0.0161 (7)	0.0095 (7)
C1	0.0385 (11)	0.0479 (12)	0.0504 (12)	0.0120 (9)	0.0201 (10)	0.0154 (10)
C2	0.0535 (13)	0.0611 (14)	0.0708 (15)	0.0250 (12)	0.0247 (12)	0.0357 (13)
C3	0.0524 (14)	0.0918 (19)	0.0571 (15)	0.0310 (14)	0.0166 (12)	0.0371 (14)
C4	0.0484 (13)	0.0759 (17)	0.0448 (13)	0.0172 (12)	0.0056 (10)	0.0110 (12)
C5	0.0435 (11)	0.0510 (12)	0.0456 (12)	0.0153 (10)	0.0109 (10)	0.0081 (10)
C6	0.0333 (10)	0.0468 (11)	0.0373 (10)	0.0147 (8)	0.0151 (8)	0.0114 (9)
C7	0.0376 (10)	0.0400 (10)	0.0377 (10)	0.0116 (9)	0.0152 (9)	0.0086 (9)
C8	0.0348 (10)	0.0388 (11)	0.0364 (10)	0.0099 (8)	0.0130 (8)	0.0066 (9)
C9	0.0375 (10)	0.0352 (10)	0.0293 (9)	0.0102 (8)	0.0122 (8)	0.0055 (8)
C10	0.0417 (11)	0.0403 (11)	0.0392 (11)	0.0068 (9)	0.0136 (9)	0.0091 (9)
C11	0.0616 (14)	0.0428 (11)	0.0529 (13)	0.0168 (10)	0.0239 (11)	0.0208 (10)
C12	0.0580 (14)	0.0552 (13)	0.0560 (13)	0.0299 (11)	0.0241 (11)	0.0279 (11)
C13	0.0376 (10)	0.0346 (9)	0.0303 (9)	0.0135 (8)	0.0137 (8)	0.0049 (8)
C14	0.0284 (9)	0.0380 (10)	0.0325 (10)	0.0119 (8)	0.0105 (8)	0.0075 (8)
C15	0.0593 (13)	0.0515 (12)	0.0343 (11)	0.0107 (10)	0.0214 (10)	0.0114 (10)
C16	0.0588 (13)	0.0451 (12)	0.0437 (12)	−0.0009 (10)	0.0259 (10)	0.0083 (10)
C17	0.0441 (11)	0.0420 (11)	0.0416 (11)	−0.0001 (9)	0.0187 (9)	0.0008 (9)
C18	0.0278 (9)	0.0383 (10)	0.0328 (10)	0.0097 (8)	0.0125 (8)	0.0050 (8)
C19	0.0290 (9)	0.0350 (9)	0.0329 (10)	0.0078 (7)	0.0118 (8)	0.0042 (8)
C20	0.0317 (10)	0.0380 (10)	0.0393 (10)	0.0133 (8)	0.0133 (8)	0.0103 (8)
C21	0.0352 (10)	0.0385 (10)	0.0405 (10)	0.0172 (8)	0.0180 (8)	0.0121 (8)
C22	0.0364 (11)	0.0719 (15)	0.0470 (12)	0.0183 (10)	0.0143 (10)	0.0058 (11)
C23	0.0567 (14)	0.107 (2)	0.0413 (13)	0.0328 (14)	0.0159 (11)	0.0042 (13)
C24	0.0662 (16)	0.102 (2)	0.0461 (13)	0.0416 (14)	0.0315 (12)	0.0171 (13)
C25	0.0450 (12)	0.0731 (15)	0.0542 (13)	0.0296 (11)	0.0289 (11)	0.0208 (11)
C26	0.0369 (10)	0.0415 (10)	0.0422 (11)	0.0184 (8)	0.0170 (9)	0.0108 (9)

### Geometric parameters (Å, °)

**Table:**

O1—C1	1.358 (2)	C8—C9	1.494 (2)
O1—H1	0.8200	C9—C10	1.390 (2)
O2—C8	1.226 (2)	C9—C13	1.398 (2)
O3—C19	1.229 (2)	C10—C11	1.371 (3)
O4—C26	1.359 (2)	C10—H10	0.9300
O4—H4	0.8200	C11—C12	1.372 (3)
N1—C7	1.279 (2)	C11—H11	0.9300
N1—N2	1.387 (2)	C12—H12	0.9300
N2—C8	1.345 (2)	C13—C14	1.502 (2)
N2—H2D	0.8600	C14—C18	1.390 (2)
N3—C13	1.335 (2)	C15—C16	1.371 (3)
N3—C12	1.340 (2)	C15—H15	0.9300
N4—C15	1.338 (2)	C16—C17	1.374 (3)
N4—C14	1.344 (2)	C16—H16	0.9300
N5—C19	1.343 (2)	C17—C18	1.384 (2)
N5—N6	1.3807 (19)	C17—H17	0.9300
N5—H5D	0.8600	C18—C19	1.498 (2)
N6—C20	1.280 (2)	C20—C21	1.461 (2)
C1—C2	1.386 (3)	C20—H20	0.9300
C1—C6	1.406 (3)	C21—C22	1.388 (3)
C2—C3	1.372 (3)	C21—C26	1.401 (2)
C2—H2	0.9300	C22—C23	1.372 (3)
C3—C4	1.371 (3)	C22—H22	0.9300
C3—H3	0.9300	C23—C24	1.383 (3)
C4—C5	1.373 (3)	C23—H23	0.9300
C4—H4A	0.9300	C24—C25	1.372 (3)
C5—C6	1.397 (3)	C24—H24	0.9300
C5—H5	0.9300	C25—C26	1.388 (3)
C6—C7	1.447 (2)	C25—H25	0.9300
C7—H7	0.9300		
			
C1—O1—H1	109.5	N3—C12—C11	123.45 (19)
C26—O4—H4	109.5	N3—C12—H12	118.3
C7—N1—N2	114.91 (16)	C11—C12—H12	118.3
C8—N2—N1	120.29 (15)	N3—C13—C9	123.44 (16)
C8—N2—H2D	119.9	N3—C13—C14	113.82 (15)
N1—N2—H2D	119.9	C9—C13—C14	122.65 (15)
C13—N3—C12	117.24 (17)	N4—C14—C18	122.77 (16)
C15—N4—C14	117.06 (16)	N4—C14—C13	115.72 (15)
C19—N5—N6	121.03 (14)	C18—C14—C13	121.31 (15)
C19—N5—H5D	119.5	N4—C15—C16	123.90 (18)
N6—N5—H5D	119.5	N4—C15—H15	118.1
C20—N6—N5	114.90 (14)	C16—C15—H15	118.1
O1—C1—C2	118.42 (19)	C15—C16—C17	118.71 (18)
O1—C1—C6	122.38 (17)	C15—C16—H16	120.6
C2—C1—C6	119.20 (19)	C17—C16—H16	120.6
C3—C2—C1	120.6 (2)	C16—C17—C18	119.02 (18)
C3—C2—H2	119.7	C16—C17—H17	120.5
C1—C2—H2	119.7	C18—C17—H17	120.5
C4—C3—C2	121.0 (2)	C17—C18—C14	118.48 (16)
C4—C3—H3	119.5	C17—C18—C19	120.92 (16)
C2—C3—H3	119.5	C14—C18—C19	120.56 (15)
C3—C4—C5	119.4 (2)	O3—C19—N5	124.31 (16)
C3—C4—H4A	120.3	O3—C19—C18	123.07 (15)
C5—C4—H4A	120.3	N5—C19—C18	112.61 (15)
C4—C5—C6	121.3 (2)	N6—C20—C21	120.59 (16)
C4—C5—H5	119.4	N6—C20—H20	119.7
C6—C5—H5	119.4	C21—C20—H20	119.7
C5—C6—C1	118.61 (17)	C22—C21—C26	118.43 (17)
C5—C6—C7	118.48 (18)	C22—C21—C20	121.77 (16)
C1—C6—C7	122.62 (17)	C26—C21—C20	119.79 (16)
N1—C7—C6	121.70 (18)	C23—C22—C21	121.58 (19)
N1—C7—H7	119.2	C23—C22—H22	119.2
C6—C7—H7	119.2	C21—C22—H22	119.2
O2—C8—N2	124.11 (17)	C22—C23—C24	119.3 (2)
O2—C8—C9	122.32 (16)	C22—C23—H23	120.3
N2—C8—C9	113.56 (16)	C24—C23—H23	120.3
C10—C9—C13	117.33 (16)	C25—C24—C23	120.5 (2)
C10—C9—C8	122.09 (17)	C25—C24—H24	119.7
C13—C9—C8	120.52 (16)	C23—C24—H24	119.7
C11—C10—C9	119.50 (18)	C24—C25—C26	120.28 (19)
C11—C10—H10	120.3	C24—C25—H25	119.9
C9—C10—H10	120.3	C26—C25—H25	119.9
C10—C11—C12	118.93 (18)	O4—C26—C25	122.21 (17)
C10—C11—H11	120.5	O4—C26—C21	117.96 (16)
C12—C11—H11	120.5	C25—C26—C21	119.82 (18)
			
C7—N1—N2—C8	178.55 (16)	C15—N4—C14—C13	176.13 (16)
C19—N5—N6—C20	178.69 (16)	N3—C13—C14—N4	−101.74 (18)
O1—C1—C2—C3	−178.8 (2)	C9—C13—C14—N4	74.8 (2)
C6—C1—C2—C3	0.5 (3)	N3—C13—C14—C18	73.3 (2)
C1—C2—C3—C4	−1.1 (4)	C9—C13—C14—C18	−110.1 (2)
C2—C3—C4—C5	1.3 (4)	C14—N4—C15—C16	−2.0 (3)
C3—C4—C5—C6	−1.0 (3)	N4—C15—C16—C17	0.6 (3)
C4—C5—C6—C1	0.4 (3)	C15—C16—C17—C18	1.7 (3)
C4—C5—C6—C7	174.46 (19)	C16—C17—C18—C14	−2.4 (3)
O1—C1—C6—C5	179.16 (18)	C16—C17—C18—C19	175.03 (17)
C2—C1—C6—C5	−0.1 (3)	N4—C14—C18—C17	1.0 (3)
O1—C1—C6—C7	5.3 (3)	C13—C14—C18—C17	−173.68 (16)
C2—C1—C6—C7	−173.96 (18)	N4—C14—C18—C19	−176.47 (16)
N2—N1—C7—C6	171.98 (15)	C13—C14—C18—C19	8.9 (2)
C5—C6—C7—N1	−173.13 (17)	N6—N5—C19—O3	−2.5 (3)
C1—C6—C7—N1	0.7 (3)	N6—N5—C19—C18	176.19 (14)
N1—N2—C8—O2	−5.5 (3)	C17—C18—C19—O3	71.2 (2)
N1—N2—C8—C9	175.49 (14)	C14—C18—C19—O3	−111.4 (2)
O2—C8—C9—C10	−137.05 (19)	C17—C18—C19—N5	−107.46 (19)
N2—C8—C9—C10	42.0 (2)	C14—C18—C19—N5	69.9 (2)
O2—C8—C9—C13	40.0 (3)	N5—N6—C20—C21	178.09 (14)
N2—C8—C9—C13	−140.91 (17)	N6—C20—C21—C22	−11.5 (3)
C13—C9—C10—C11	−1.0 (3)	N6—C20—C21—C26	169.45 (17)
C8—C9—C10—C11	176.21 (17)	C26—C21—C22—C23	−1.0 (3)
C9—C10—C11—C12	−1.8 (3)	C20—C21—C22—C23	−180.0 (2)
C13—N3—C12—C11	−0.5 (3)	C21—C22—C23—C24	−0.6 (4)
C10—C11—C12—N3	2.7 (3)	C22—C23—C24—C25	1.0 (4)
C12—N3—C13—C9	−2.6 (3)	C23—C24—C25—C26	0.3 (4)
C12—N3—C13—C14	173.98 (16)	C24—C25—C26—O4	179.0 (2)
C10—C9—C13—N3	3.3 (3)	C24—C25—C26—C21	−1.9 (3)
C8—C9—C13—N3	−173.91 (16)	C22—C21—C26—O4	−178.60 (18)
C10—C9—C13—C14	−172.94 (16)	C20—C21—C26—O4	0.4 (3)
C8—C9—C13—C14	9.8 (3)	C22—C21—C26—C25	2.2 (3)
C15—N4—C14—C18	1.2 (3)	C20—C21—C26—C25	−178.76 (17)

### Hydrogen-bond geometry (Å, °)

**Table:**

*D*—H···*A*	*D*—H	H···*A*	*D*···*A*	*D*—H···*A*
N5—H5D···O2	0.86	2.15	2.962 (2)	157
N2—H2D···N4^i^	0.86	2.17	2.985 (2)	159
O4—H4···O3^ii^	0.82	1.92	2.736 (2)	172
O1—H1···N1	0.82	1.95	2.663 (2)	145
C10—H10···Cg3^iii^	0.93	2.76	3.458 (2)	133
C11—H11···Cg4^iv^	0.93	2.73	3.588 (2)	154

Symmetry codes: (i) −*x*+1, −*y*+2, −*z*+1; (ii) *x*+1, *y*, *z*; (iii) −*x*+2, −*y*+2, −*z*+1; (iv) −*x*+1, −*y*+2, −*z*.
